# Physical Activity and Quality of Life in Malaysian Breast Cancer Survivors: A Longitudinal Study

**DOI:** 10.1200/GO.22.37000

**Published:** 2022-05-05

**Authors:** Tania Islam, Lee Yi Lin, Mahmoud Danaee, Nur Aishah Taib

**Affiliations:** ^1^ University of Malaya, Kuala Lumpur, Malaysia

## Abstract

Regular physical activity (PA) after a diagnosis of breast cancer(BC) is associated with reduced morbidity and a higher quality of life (QOL). Investigate the association of PA and QOL among Malaysian BC survivors in this prospective cohort study.

**METHODS:**

A total of, 133 BC women were interviewed at baseline, one year, and three-year after diagnosis of BC in a tertiary referral center in Klang Valley. The Global Physical Activity Questionnaire and the European Organization for Research and Treatment of Cancer Core Quality of Life Questionnaire were used to measure PA and QOL of the survivors. A generalized estimating equation was performed to examine the association of PA and QOL longitudinally.

**RESULTS:**

The mean age was 56.89 (± 10.56) years old, and the majority were Chinese (50.4%). At baseline, 48.1% of patients were active and it declined to 39.8% at one year, and 35.3% at third year. Three years after diagnosis (Adjusted Odds Ratio [AOR]: 1.74, *P* = .021), being underweight (AOR: 3.43, *P* = .004) and being Malay (AOR: 1.86, *P* = .042) were found to be significant associating factors with inactivity. Overall, the QOL was observed to decrease over the years. Global health status, physical and cognitive functioning had a significant declining trend from baseline to three years (*P* = < 0.001, *P* = < 0.001 and *P* = .001 respectively). For symptom scales, patients experienced more fatigue over time too (*P* = .006). When analysing the association of PA and QOL, being active significantly improve role functioning (*P* = .022). At one year, being active also significantly reduced constipation symptoms (*P* = .048).

**CONCLUSION:**

The findings showed that BC survivors in Malaysia had inadequate PA levels at diagnosis and further decreased over time. Their QOL had a declining trend, but PA was found to improve constipation symptoms and role functioning. Thus, it is essential to promote PA throughout their follow-ups with more focus on Malay ethnicity.

